# A numerical study on CO migration after blasting in high-altitude tunnel by inclined shaft

**DOI:** 10.1038/s41598-022-18995-y

**Published:** 2022-08-29

**Authors:** Bo Wu, Rui Zhao, Guowang Meng, Shixiang Xu, Weixing Qiu, Huihao Chen

**Affiliations:** 1 College of Civil Engineering and Architecture, Guangxi University, 100 University Road, Nanning, 530004 Guangxi China; 2 School of Civil and Architectural Engineering, East China University of Technology, Nanchang, 330013 Jiangxi China; 3 School of Architectural Engineering, Guangzhou City Construction College, Guangzhou, 510925 Guangdong China

**Keywords:** Civil engineering, Fluid dynamics

## Abstract

On the western plateau of China, ventilation problems brought on by low atmospheric pressure must be overcome. And CO migration after blasting in high-altitude tunnel by inclined shaft has become a significant scientific issue. In this study, the Computational Fluid Dynamics (CFD) method was used to analyze the flow field characteristics at the junction of the inclined shaft and tunnel. In addition, the effects of different fan opening modes and different initial CO concentration distributions on the ventilation were discussed. The simulation results showed that the main difference in the ventilation wind field was reflected in the position of the vortex region due to the different fan opening modes. Meanwhile, various initial CO concentration distributions showed different migration when there was no air volume difference between the left and right tunnels. Eliminating vortex zones and fully using high velocity airflow could improve relative ventilation efficiency by at least 18%. CO would accumulate in the opposite direction of the tunnel if only one of the fans was turned on. Therefore, a two-stage ventilation scheme was proposed, and the energy consumption was reduced by at least 33%. This research can provide guidance on high-altitude tunnel construction with multiple working faces to improve ventilation efficiency and reduce energy consumption.

## Introduction

Western China is distinguished by its high elevation, extreme cold, and low atmospheric pressure. Meanwhile, the tunnel blasting will generate a large amount of carbon monoxide (CO). Hemoglobin (Hb) has a high affinity for CO, which will significantly reduce the ability of blood to transport oxygen. It is even more fatal in the harsh environment of the highland plateau^1^. In order to guarantee the occupational health of workers and ensure the smooth construction of a high-altitude tunnel, it is necessary to study the CO propagation law of high-altitude tunnel in more depth.

During tunnel construction, forced ventilation is still the main mode of diluting toxic gases. De Souza and Katsabanis^2^ used an explosion gas diffusion model to determine the safe re-entry time, taking into account the dilution requirements of hazardous gases. To reduce toxic gas concentrations below the concentration limit as soon as possible and cut ventilation costs, the flow field characteristics in a tunnel must be studied for ventilation optimization. Parra et al.^3^ investigated three different types of ventilation systems and observed that the air duct layout directly affected the airflow field. Kurnia et al.^4^ introduced a new intermittent air ventilation system in order to save energy. However, some ventilation dead zones exist in tunnels, such as the cross-aisle of a twin-tunnel, where dangerous gases are more concentrated. It has been demonstrated that the application of a jet fan can solve this problem^5^. Furthermore, different jet fan parameters have different effects on the improvement of ventilation efficiency^6–10^. In addition, air curtain technology is gradually being implemented in tunnel ventilation. It has the ability to control dust or blasting fumes in a specific area, which are quickly exhausted through the duct^11^. Many researchers have investigated the flow field characteristics and optimal parameters of air curtain ventilation^12–14^. What's more, it is widely accepted that all ventilation ducts leak to some extent, and duct efficiency can be used to evaluate the impacts of leakage rate on ventilation systems^15,16^. Wang et al.^17^ established a three-dimensional model with CFD to analyze the leakage rate. The simulation results revealed that the pressure and leakage volume have an effect on the leakage rate along the tunnel.

But the increase in altitude brings greater challenges to tunnel ventilation. The required airflow volume, supply volume of fan, and flow field characteristics in plain areas are no longer applicable to plateau areas. First of all, oxygen deprivation will aggravate insufficient combustion of the engine and increase the discharge of hazardous gases. In general, the Real Driving Emissions (RDE) test is the main research method to study exhaust emissions^18^. Ramos et al.^19^ conducted field tests with three different fuels and comprehensively studied the effects of altitude, alternative fuels, and driving conditions on exhaust emissions. The results indicated that at high altitude, the nitrogen oxide (NOx) emissions were about ten times higher than the limits set by European standards. Wang et al.^20^ observed that the CO, PN, and NOx emissions all increased with altitude, while the NOx emissions declined when the altitude exceeded 2990 m. Corresponding to the CO altitude coefficient, the NOx altitude coefficient can be used to reflect the impact of altitude on NOx emission factors. According to the comparison, CO is more significantly affected by altitude^21^. Secondly, the plateau environment will exert a great influence on many physiological systems of the human body, reducing average labor capacity and lowering resistance to toxic gases. Workers are more prone to dizziness and even poisoning^22–24^. The well-known Coburn–Forster–Kane (CFK) equation describes a functional relationship between CO concentration in the environment and carboxyhemoglobin (COHb) concentration in the human body, laying the theoretical solid foundations for the study of the CO concentration limit^25^. Furthermore, a drop in air density will have an effect on the normal operation of the fan in the plateau area^26^. As a consequence, the fan must be changed to satisfy the ventilation needs of the high-altitude environment. Under the high-altitude tunnel construction, the space–time evolution of toxic gases or dust will change, which directly affects the ventilation layout. The characteristics of smoke transport in tunnel fires at high altitudes have been thoroughly investigated and studied^27–29^, which may provide research ideas for high-altitude tunnel ventilation after blasting. Huang, Shen, Wang and Liao^30^ studied the CO migration law after blasting in a plateau mine using a CFD model, pointing out that the ventilation time required to dilute CO in a low-altitude location is obviously less than in a high-altitude area. Feng et al.^31^ used numerical simulations to establish a set of CO concentration functions under diverse altitude situations, which were verified through field data.

In conclusion, many scholars have conducted research on high-altitude tunnel ventilation and achieved fruitful results. However, how to optimize the ventilation layout in the face of more complex construction conditions remains a significant problem that requires further study. In order to shorten the construction period, the inclined shaft is often set up in a long tunnel to add more working faces. And the airflows from the left and right tunnels will converge at the junction of the tunnel and inclined shaft. Once vortex zones are generated, the ventilation efficiency will decrease. The purpose of this study is to explore the CO migration in high-altitude tunnels by inclined shaft and reveal the influence of different ventilation arrangements.

## Methodology

### Mathematical models

In fluid flow, the conservation of mass, the conservation of momentum, and the conservation of energy should be followed. And if there are a variety of species, each species should also conform to the conservation of mass. Laminar flow and turbulent flow are identical in the form of the general governing equation, with variables possessing different expressions. Furthermore, the turbulence-transport equation must be included in the turbulence calculation. The governing equation of laminar flow is as follows.

Mass conservation equation or continuity equation^31^:

1 $$\frac{\partial \rho }{\partial t}+\nabla \left(\rho u\right)=0$$

Momentum conservation equation:

2 $$\frac{\partial }{\partial t}\left(\rho u\right)+\nabla \left(\rho uu\right)=-\nabla p+\nabla \tau +F$$

Energy conservation equation:

3 $$\frac{\partial }{\partial t}\left(\rho T\right)+\nabla \left(\rho uT\right)=\nabla (\frac{\omega }{{c}_{p}}\mathrm{grad}T)+{S}_{T}$$

Species mass-conservation equation:

4 $$\frac{\partial }{\partial t}\left(\rho {c}_{s}\right)+\nabla \left(\rho u{c}_{s}\right)=\nabla ({D}_{s}\mathrm{grad}(\rho {c}_{s}))$$

where $\rho$ represents the gas density, kg/m^3^; $t$ represents the time, s; $p$ represents the pressure, Pa; *u* = (*v*_*x*_, *v*_*y*_, *v*_*z*_) represents the velocity vector, m/s; τ represents the stress tensor; *T* represents the temperature, K; $F=\left({F}_{x},{F}_{y}{,F}_{z}\right)\mathrm{ is}$ the force, N; $\omega$ is the total heat transfer coefficient, W/(m $\mathrm{K}$); ${c}_{p}$ represents the specific heat capacity, J/(kg $\mathrm{K}$); ${S}_{T}$ represents the viscous dissipation term; ${c}_{s}$ represents the mass fraction; ${D}_{s}$ represents the diffusion coefficient, m^2^/s.

The standard *k*–*ε* model is established for the flow with fully developed turbulence, but is not suitable for strong swirling flow or flow along the curved wall surface. Yakhot and Orzag^32^ first proposed the RNG *k*–*ε* model, which is effective to predict complex turbulent flows with high strain rates. It has been applied well in pollutant dispersion^33–36^. The equations of *k* and *ε* are as follows:

5 $$\frac{\partial }{\partial t}\left(\rho k\right)+\nabla \left(\rho ku\right)=\nabla \left[\left(\mu +\frac{{\mu }_{t}}{{\sigma }_{k}}\right)\nabla k\right]+{G}_{k}-\rho \varepsilon $$

6 $$\frac{\partial }{\partial t}\left(\rho \varepsilon \right)+\nabla \left(\rho \varepsilon u\right)=\nabla \left[\left(\mu +\frac{{\mu }_{t}}{{\sigma }_{\varepsilon }}\right)\nabla \varepsilon \right]+{C}_{1\varepsilon }\frac{\varepsilon {G}_{k}}{k}-{C}_{2\varepsilon }\frac{{\varepsilon }^{2}}{k}-{R}_{\varepsilon }$$

where $k$ represents the turbulent kinetic energy, ${m}^{2}/{s}^{2}; {G}_{k}$ represents the production due to mean velocity shear; $\varepsilon$ represents the dissipation rate of turbulent kinetic energy, ${m}^{2}/{s}^{3}; \mu$ and ${\mu }_{t}$ are laminar and turbulent viscosity, $\mathrm{pa s}$, ${\mu }_{t}=\rho {C}_{\mu }{k}^{2}/\varepsilon$; ${C}_{1\varepsilon }$, ${C}_{2\varepsilon }$, ${C}_{\mu }$, ${\sigma }_{k}$ and ${\sigma }_{\varepsilon }$ are constants with ${C}_{1\varepsilon }=1.42$, ${C}_{2\varepsilon }=1.68$, ${C}_{\mu }=0.0845$, ${\sigma }_{k}={ \sigma }_{\varepsilon }=0.7179; {R}_{\varepsilon }$ represents an additional term.

No matter it is the standard *k–ε* model, the RNG *k–ε* model, or the Realizable *k–ε* model, they are only effective for fully developed turbulence, that is, the above three models are all high Reynolds number models, which can only solve flows in the core region of turbulence. While in the wall region, the flow situations vary greatly. Especially in the viscous sublayer, the flow is almost laminar, and the pulsation effect of turbulence is less than that of molecular viscosity. The wall function is usually applied to this zone.

### Gas parameters

Due to the compressibility of air, there is a nonlinear relationship between atmospheric pressure and altitude. Meanwhile, the air density is reduced. Ignoring the effects of latitude, longitude, and season, the atmospheric pressure and gas density varying with altitude can be calculated by the following formulas^30^:

7 $$P=101325\cdot {\left(1-\frac{H}{44329}\right)}^{5.2559}$$

8 $$\rho ={\rho }_{0}\cdot \frac{P}{101325}\cdot \frac{273.15}{T}$$

where $P$ represents the atmospheric pressure at different altitude, Pa; $H$ represents the altitude, m; ${\rho }_{0}$ represents the air density under standard conditions, 1.293 kg/m^3^; $T$ represents the temperature at different altitude, K.

A throwing area of smoke will be formed in the tunnel after blasting. A large number of gaseous products will be produced in this process. The blasting fumes contains a variety of components, most of which are toxic and harmful to the human health. In this paper, CO of blasting fumes is taken as the research object. It is assumed that CO is evenly distributed in the throwing area, and the mass concentration can be calculated as follows:

9 $${L}_{0}=15+\frac{m}{5}$$

10 $$C=\frac{m\cdot q\cdot {M}_{CO}}{{L}_{0}\cdot A\cdot {M}_{air}}$$

11 $${C}_{m}=\frac{{C}_{v}\cdot {M}_{CO}}{22.4}\cdot \frac{P}{101325}\cdot \frac{273.15}{T}$$

where $C$ represents the mass fraction of CO; $m$ represents the explosive initiation quantity, kg; $q$ represents the volume of CO generated by explosive per unit mass, 0.04 m^3^/kg; ${M}_{CO}$ represents the molar mass of CO, 28 g/mol; ${M}_{air}$ represents the molar mass of air, 29 g/mol; ${L}_{0}$ represents the throwing length of smoke, m; $A$ represents the cross-sectional area of tunnel, m^2^; ${C}_{m}$ represents the mass concentration of CO as initial value, mg/m^3^; ${C}_{v}$ represents the parts per million of CO.

## Numerical model

### Numerical scenarios

The Kangding tunnel is one of the dominant engineering projects on the Sichuan-Xizang railway, which is located in the section between Kangding and Huojiazhong. The total length of the tunnel is 20,793 m, and the altitude is 3700 m. Because it is located in a semi-arid climate zone of the plateau, with a long and cold winter, frequent low temperature and frost disasters, and serious freeze–thaw diseases, effective thermal insulation measures must be taken in the process of construction. Affected by the ultra-high altitude, the atmospheric oxygen content in the low-pressure environment is only about 60% of that at sea level. Meanwhile, the oxygen content in the tunnel is lower than that outside. Construction workers and mechanical equipment have suffered significant losses in work efficiency in an oxygen-deficient environment. The construction of the 2# inclined shaft in Kangding Tunnel is divided into two stages: the first stage is the construction of the inclined shaft, and the second stage is the construction of the left and right tunnels at the same time. Forced ventilation is used in both stages to dilute harmful gases. This paper mainly studies the ventilation arrangement of the second stage.

Because of the complex working conditions that exist during the tunnel's actual construction, there are numerous unforeseen aspects in the real-world situation that can't be fully considered by computer numerical simulation. As a result, several unimportant parameters that have no bearing on the final simulation results can be overlooked. For the sake of numerical calculation, the following assumptions are proposed: (1) gas is made up of a large number of molecules. A single molecule's flow state is defined by discontinuity in space and randomness in time. The gas is characterized by continuity and certainty in space and time when the number of molecules reaches a specific level. Because the simulation research in this work is based on Fluent, the air in the tunnel should be treated as a continuous medium before the simulation. (2) Because all fluids are compressible, their density varies with temperature and pressure. The airflow in the tunnel, on the other hand, is a low-speed fluid that is viscous and incompressible. (3) In the tunnel, the airflow field maintains a constant temperature, and there is no heat transfer between the wall and the environment. (4) During the diffusion phase, the gases are merely air and CO, and no chemical reactions occur.

**Figure 1:**
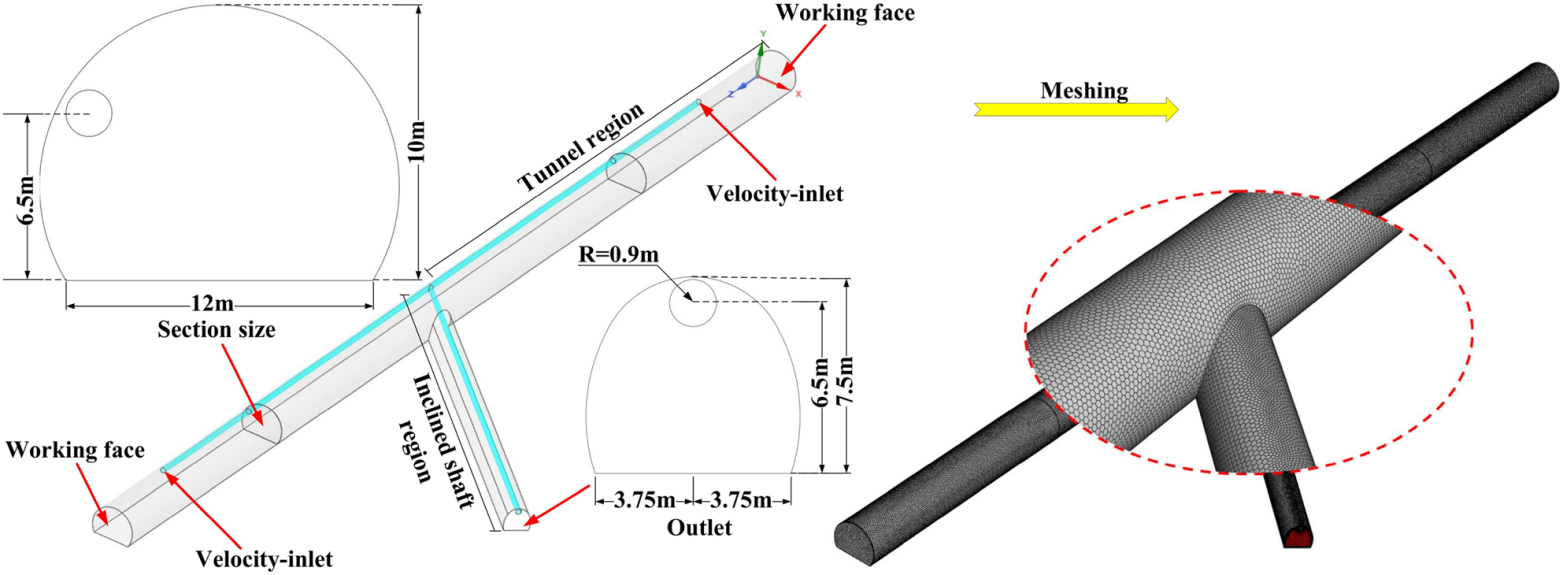
Physical model and mesh of the tunnel.

The ANSYS 2020R2 (https://www.ansys.com/products/3d-design/ansys-spaceclaim) was used to establish a 3D model. The tunnel direction is along Z-axis, and the right working face is located in the X–Y plane. The sectional size of the tunnel and the inclined shaft are 128 m^2^ and 52 m^2^, and 170 m behind each working face are selected for calculation. The distance between the air duct and the working face is 35 m, and the diameter of the air duct is 1.8 m. The angle formed by the inclined shaft and the left tunnel is 45°. Considering the actual situation of this numerical simulation, it takes a long time to divide structured grid by ICEM. Although the efficiency of the calculation can be substantially increased by using the unstructured mesh, the quality of the tetrahedral mesh is poor, and the solution is easily diverged. Therefore, Fluent-Meshing was selected to divide the mesh, which not only ensure the mesh quality, but also greatly saved the time of grid generation. Polyhedra was selected as the mesh types. The physical model and the mesh of the tunnel are as shown in Fig. 1.

The boundary conditions and initial conditions are called the solution conditions. The unique solution of the flow field exists, only when the boundary conditions and initial conditions are determined. After the mesh was imported into Fluent software, the corresponding boundary conditions need to be set as follows: (1) both inlets were set as the Velocity-inlet, and the velocity magnitude was 20 m/s. (2) The outlet was set as the Pressure-outlet. (3) The working face and the interior wall of the tunnel are both fixed walls, and were set as the Wall boundary.

Studying the influence of stable flow field on ventilation effect after blasting plays an important role in determining the ventilation scheme for high-altitude tunnel construction, improving ventilation efficiency and reducing energy consumption. A steady-state simulation can be used first to consider the influence of a stable flow field on tunnel ventilation. The values of all residuals decreased rapidly to less than $1\times {10}^{-3}$, indicating convergence of calculation and providing better initial conditions for transient simulation. Solution parameters in numerical simulation are shown in Table 1.

**Table 1:** Solution parameters in numerical simulation.

Parameter type	Parameter	Values and settings
Gas parameter	Air density/(kg/m^3^)	0.842
	CO density/(kg/m^3^)	0.814
	Temperature/K	265.4
	Atmospheric pressure/(Pa)	64,078
	Dynamic viscosity coefficient of air/(Pa⋅s)	1.68 × 10^−5^
Blasting parameter values	Throwing length of blasting fume/(m)	82
	Mass fraction of CO/(%)	0.12
	Mass concentration of CO/(kg/m^3^)	0.000976
Solver settings	Time	Steady (before blasting) Transient (after blasting)
	Solver type	Pressure-based
	Turbulence model	RNG *k–ε*
	Velocity of inlet/(m/s)	20
	Turbulent intensity/(%)	2.64
	Hydraulic diameter/(m)	1.8
	Solution Methods	SIMPLEC
	Time step/(s)	0.1

### Verification of the modelling method

**Figure 2:**
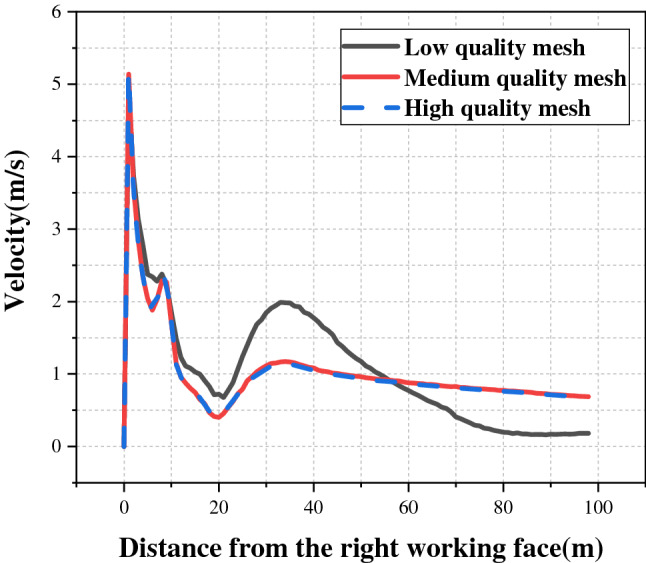
Mesh independence analysis.

When CFD is used to analyze flow fields and gas diffusion, poor mesh quality will directly affect the accuracy and reliability of simulation, so it is necessary to test the independence of the mesh. Fluent-Meshing was used to divide the model into three different quality grids, including low-quality grids (1,982,150 cells), medium-quality grids (3,075,091 cells) and high-quality grids (3,472,652 cells). The average air velocity was selected as the index of mesh independence detection, and Fluent was imported to calculate the velocity distribution of the three grids. Figure 2 shows that the average air velocity of the medium-quality grids and the high-quality grids was very close, indicating that mesh independence has been achieved. In this study, medium-quality grids were selected to ensure computational efficiency and accuracy.

**Figure 3:**
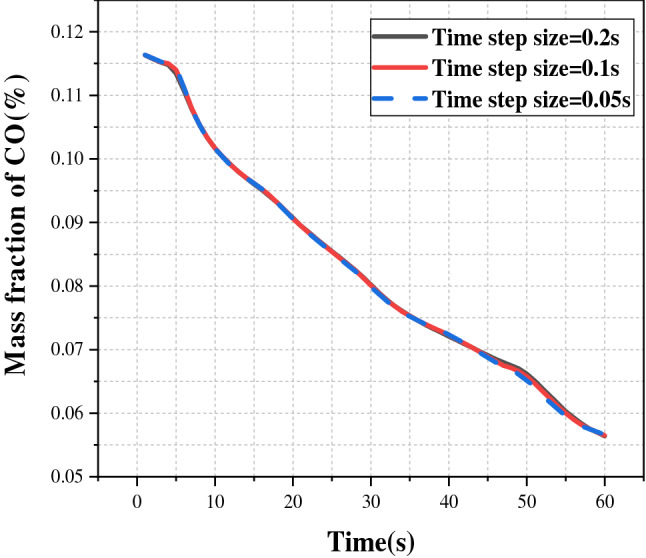
Verification of the modeling method.

Aside from ensuring that the calculation results are independent of meshing for the transient simulation, it is also necessary to ensure that the time step does not affect the final calculation results. If the time step is too large, the calculation deviation may be significant, resulting in the failure to show the true physical change law. Therefore, the section Z = 10 m was selected, and the average mass fraction of CO in this section was calculated. Three different types of time steps were chosen: 0.2 s, 0.1 s, and 0.05 s. The average mass fraction of CO in this section within 60 s is shown in Fig. 3. The diagram shows that the three curves were fairly close, indicating that the time step had little effect on the calculation results. For the study, the time step was set to 0.1 s to ensure calculation efficiency.

Since the project is still in the preparatory stage, the corresponding field data cannot be obtained. This study serves as preliminary research to provide corresponding guidance and suggestions for practical construction. The turbulent jet theory is widely studied and verified by experimental data since last century^37–39^. To further verify the accuracy of the model, the simulation values of air velocity in the fully developed flow region were compared with theoretical calculation values, as shown in Table 2. Although there was a certain difference between the simulation value and the empirical formula value, but the reduction trend was the same, and the absolute error also fluctuated around 1.8 m/s. Considering the influence of the velocity gradient, truncation error, and the change of gas physical parameters in this numerical simulation, the modeling method used in this study is feasible. The axial velocity in the fully developed flow region can be calculated as follows:

12 $$\frac{{v}_{m}}{{v}_{0}}=\frac{0.48}{\frac{\alpha S}{\sqrt{2}{d}_{0}}+0.147}$$

where ${v}_{m}$ represents axial velocity of the turbulent jet, m/s; ${v}_{0}$ represents the velocity of air outlet, m/s; $\alpha$ represents the turbulence coefficient, 0.08 for round pipe; $S$ represents the distance from the ventiduct mouth, m; ${d}_{0}$ represents the diameter of the duct, m.

**Table 2:** Comparison between simulation values and theoretical values.

Distance from the duct (m)	30	29	28	27	26	25	24	23	22	21
Simulation values (m/s)	10.53	10.89	11.13	11.49	11.79	12.05	12.52	12.86	13.23	13.69
Theoretical values (m/s)	8.81	9.07	9.35	9.64	9.96	10.29	10.65	11.04	11.45	11.90
Absolute error (m/s)	1.72	1.82	1.78	1.85	1.83	1.76	1.87	1.82	1.78	1.79

## Results and discussion

In the second stage of construction, fresh air is forced from the ventiduct mouth to the working face by an axial fan in both directions to dilute the harmful gases. The polluted airflow is eventually discharged from the inclined shaft. However, air flow and harmful gas diffusion are different from those in a single tunnel ventilation system due to the confluence of airflow in two directions at the junction area of the inclined shaft and tunnel. This paper has chosen five working conditions for analysis in order to study the migration of CO in high-altitude tunnels by inclined shaft as comprehensively as possible. Case 1: Both directions of the tunnel carry out drilling and blasting construction, and the fans in both directions work at the same time. Case 2: Only the left tunnel carries out drilling and blasting construction, but the fans in both directions work at the same time. Case 3: Only the right tunnel carries out drilling and blasting construction, but the fans in both directions work at the same time. Case 4: Only the left tunnel carries out drilling and blasting construction, and the fan in this direction works. Case 5: Only the right tunnel carries out drilling and blasting construction, and the fan in this direction works.

According to China's national code, "Safety regulations for blasting", appropriate ventilation should be carried out following blasting construction. The construction staff are authorized to access the blasting operation site after ensuring that the air quality of the subterranean blasting site is certified, the ventilation is enough, and the waiting time is greater than 15 min. Therefore, this paper mainly takes the breathing height y = 1.5 m as the research plane to analyze the flow field distribution and CO concentration distribution in the tunnel.

### Analysis of airflow field distribution

**Figure 4:**
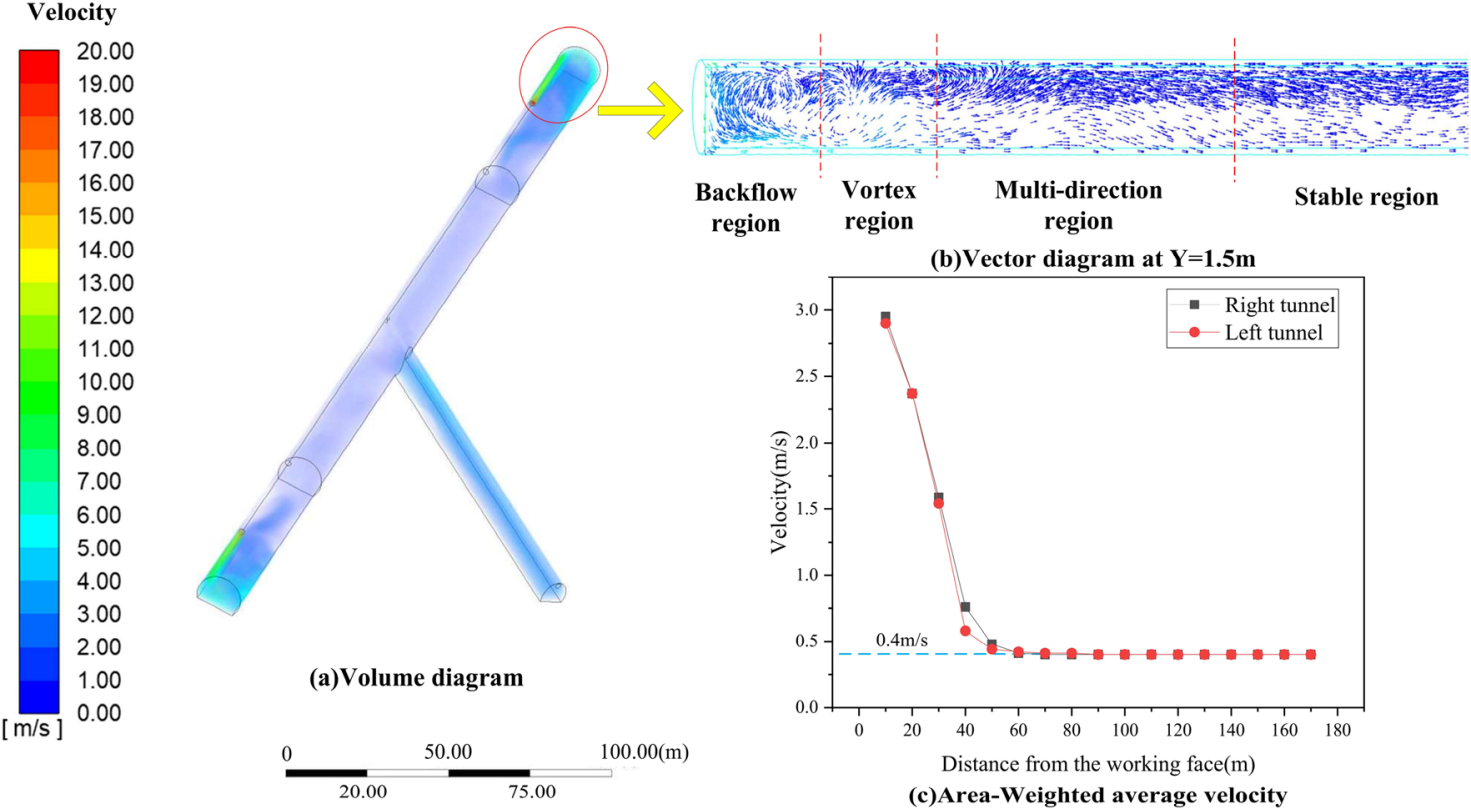
Airflow field of tunnel region.

The ventilation on both sides is not completely symmetrical because the inclined shaft does not intersect the tunnel vertically. Therefore, the ventilation of the left and right tunnels when they are constructed separately should be analyzed and compared to the ventilation of simultaneous construction. The distribution of airflow field in tunnel was shown in Fig. 4a.

There are just three ventilation flow fields for the five construction conditions listed above. For study and discussion, any type of ventilation flow field can be divided into tunnel region and inclined shaft region, as shown in Fig. 1.

#### Analysis of tunnel region

As shown in Fig. 4b, the typical characteristics of wall jets were displayed near the working face. The flow development region gradually expanded from the outlet of the air duct, and the surrounding air was drawn into the jet flow. When colliding with the working face in the tunnel, the jet flow moved in the opposite direction because of the obstruction of the working face, thereby creating a backflow area. Due to the entrainment effect of the high-speed jet, it would entrain the low-speed air around it, resulting in the formation of a vortex area between the ventiduct mouth and the working face. And the reverse flow would also be affected in this area. Because of the swirling motion of particles in the vortex region, the diffusion of pollutants would be hindered. When the distance between the working face and the ventiduct mouth exceeded 35 m, the jet flow would no longer suck in the surrounding air. And overall air flow tends to be stable as the distance from the working face increases. This is in accordance with the previous research^40^.

In order to analyze the variation of area-average wind speed with distance more directly, 17 sections were evenly obtained every 10 m along both the left and right tunnels. The fluid viscosity converted kinetic energy to internal energy, resulting in a gradual decrease in wind speed with distance, which dropped to about 0.40 m/s at 70 m away from the working face, as shown in Fig. 4c. The two average wind speed curves were essentially identical, indicating that the simulation was reliable.

#### Analysis of inclined shaft region

The largest variation between the three ventilation flow fields is in the inclined shaft region, due to the varied opening conditions of the fans in the left and right tunnels.

**Figure 5:**
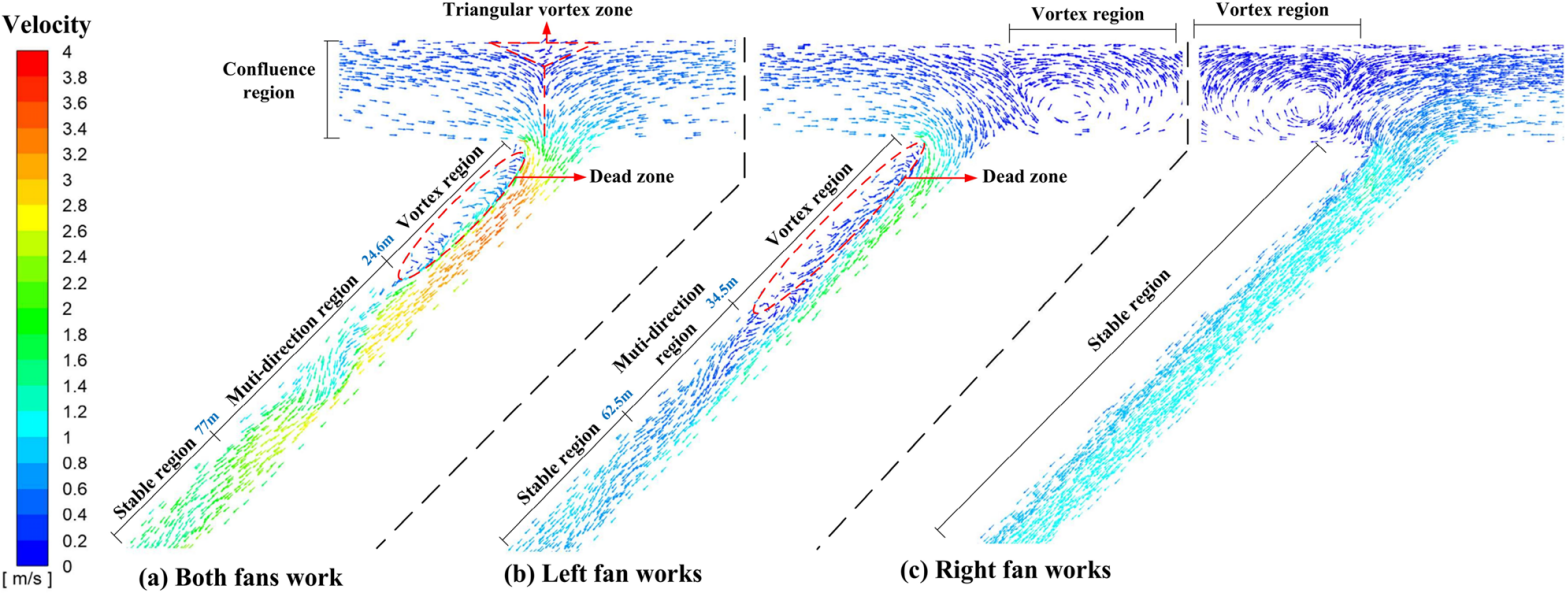
Airflow field of inclined shaft region.

When the fans of the left and right tunnel are turned on at the same time, the ventilation flow field in the inclined shaft area can be divided into 4 regions: the confluence region, vortex region, Muti-direction region and stable region. The flow field in the inclined shaft area is shown in Fig. 5a, and the following conclusions can be drawn: (1) Because of the low velocity of the flow field, no visible vortex was formed at the confluence of the inclined shaft and the tunnel, but there was an obvious dividing line when the left and right opposite air flows converged. It was divided by red dashes in the diagram. (2) As the airflow moved towards the inclined shaft, it split from the side wall and formed a triangular low-velocity vortex zone. (3) The air volume was equal in both directions when the two fans maintained the same power. However, the width of airflow entering the inclined shaft from the left tunnel was slightly smaller than that from the right tunnel. It was considered that the angle between the left tunnel and the inclined shaft was an acute angle, hence the momentum change of the left airflow into the inclined shaft was greater than that of the right airflow. Therefore, the energy loss of the left airflow was greater than that of the right airflow. (4) The airflow rate increased as it entered the inclined shaft from the tunnel. The reason was that the area of the inclined shaft section was smaller than that of the tunnel section. When the volume of airflow remained constant, the section area decreased and the speed increased. Combined with conclusion 2, the velocity increase amplitude of the left airflow was greater than that of the right airflow, which was consistent with the velocity distribution image. (5) In addition, it was worth noting that there was a ventilation dead zone with a length of about 24.6 m on the left side of the inclined shaft, where the main stream was separated from the wall, forming a vortex zone between the main stream and the wall due to the inertia action. The vortex zone increased the turbulence of the fluid. Meanwhile, the mass exchange between the vortex region and the mainstream region would continue, and the moving particles of the vortex would be carried downstream by the mainstream, which intensified the turbulence intensity of the mainstream within a certain range of the downstream and further increased the energy loss. (6) The adverse pressure gradient occurred as a result of boundary layer separation, resulting in airflow moving to the left side of the inclined shaft. The vortex area vanished when the airflow reached the left wall of the inclined shaft. When the airflow was blocked by the left wall of the inclined shaft, it shifted to the right side and intersected with the main stream again along the inclined shaft, forming a multi-directional area with a length of about 52.4 m.

When only the fan of the left tunnel was turned on, the flow field in the inclined shaft area is shown in Fig. 5b, and the following conclusions can be drawn: (1) The section width of the tunnel was greater than that of the inclined shaft. As a result, when the airflow in the left tunnel moved to the inclined shaft, part of the airflow would be blocked by the wall of the right tunnel and create a vortex area in the right tunnel. (2) Similar to the case when the left and right fans were turned on at the same time, a ventilation dead zone would be formed on the left side of the inclined shaft. (3) For the reason that the air volume of a single fan was less than that of two fans, the air velocity increment after the airflow entering the inclined shaft was greatly reduced, and the overall air flow tended to be stable faster. However, the adverse pressure gradient was also small, which made the vortex region longer than that shown in Fig. 5a.

When only the fan of the right tunnel was turned on, the flow field in the inclined shaft area is shown in Fig. 5c, and the following conclusions can be drawn: (1) Similar to the case when only the fan of the left tunnel was turned on, part of the airflow would be blocked by the wall of the left tunnel and create a vortex area in the left tunnel when the airflow in the right tunnel moved to the inclined shaft. (2) The angle formed by the right tunnel and the inclined shaft was obtuse. As a result, the airflow entered the inclined shaft more smoothly. Furthermore, because there was no separation between the airflow and the wall, there was no noticeable vortex area in the inclined shaft. (3) The overall airflow in the inclined shaft remained stable, with no evidence of velocity gradient stratification.

### Analysis of CO concentration distribution

The distribution of the airflow field has a significant impact on the distribution of the CO concentration field. However, the migration process is dynamic and complex. It is difficult to establish the CO distribution law over time by analyzing the air flow field alone. Therefore, further discussion of the CO concentration field is required.

#### Analysis of case 1

**Figure 6:**
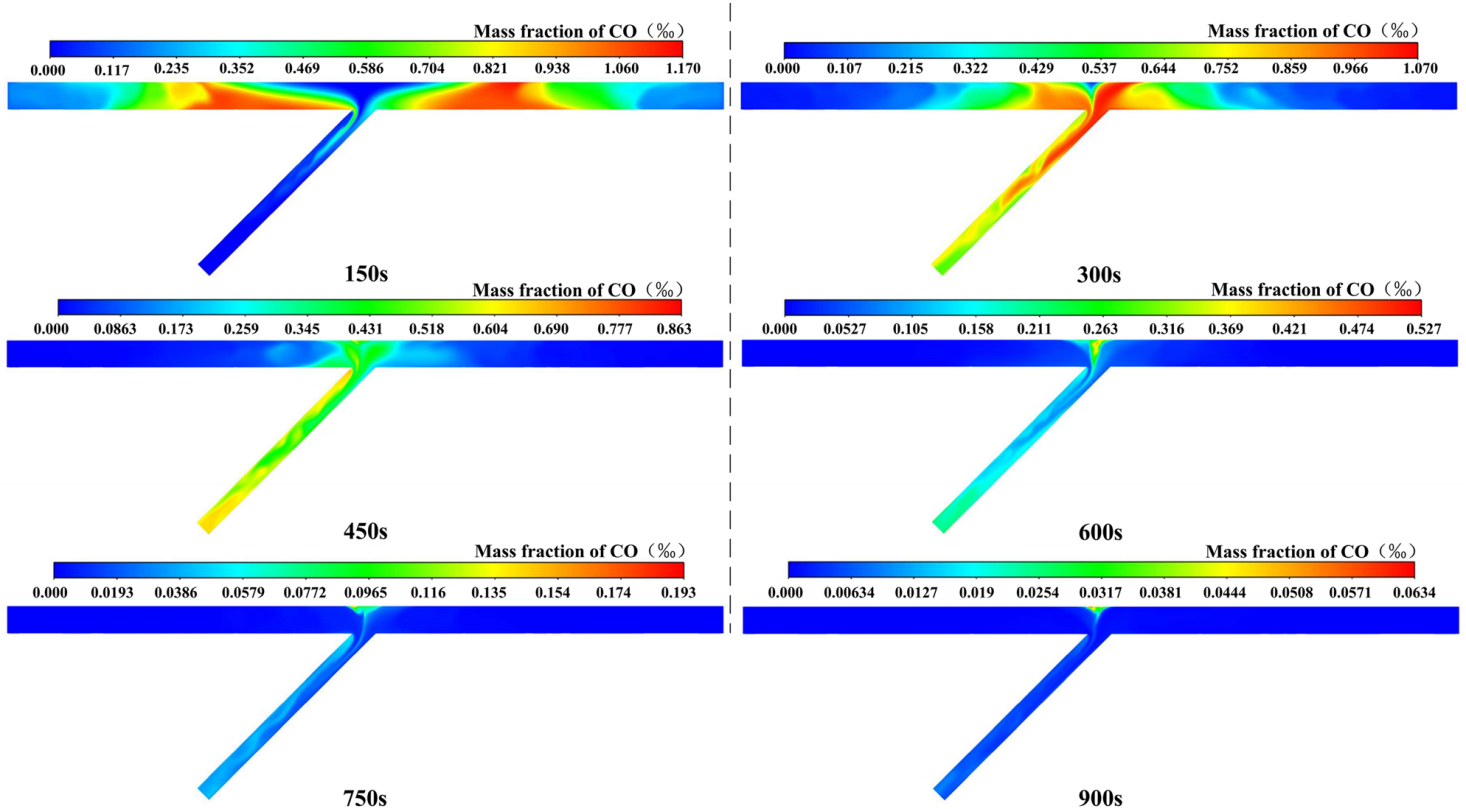
CO distribution of case 1.

The CO distribution over time of case 1 is shown in Fig. 6. The following conclusions can be drawn: (1) After ventilating for 150 s, the CO migrated towards the exit of the inclined shaft and presented the coupling effect of migration and diffusion. Meanwhile, the CO zone was deformed gradually under the effect of air flow and was continuously elongated in the tunnel region, in which a concentration peak of CO could be found. Besides, part of the CO in the left tunnel first entered the inclined shaft and then extended into a slender strip, with the extended length being greater than that of the right tunnel. (2) After ventilating for 300 s, the CO was effectively diluted near the working face. A clear boundary could be found between the air masses under the effect of the flow field in the confluence region. Note that the CO had not yet diffused into the triangular vortex zone. In addition, the airflow in both directions was fully mixed in the vortex region of the inclined shaft, forming a concentration peak. The CO gradually diffused into the ventilation dead zone on the left side of the inclined shaft. Moreover, the CO was shown to have a wavy shape in the multi-direction region of the inclined shaft. And the CO was gradually uniformly distributed with the increasing ventilation distance. (3) After ventilating for 450 s, the peak value of CO mass fraction had reached the outlet of the inclined shaft. However, due to the influence of the ventilation dead zone on the left side of the inclined shaft, the concentration peak also appeared in this area. (4) After ventilating for 600 s, the CO would stay in the triangular vortex zone and the ventilation dead zone on the left side of the inclined shaft, and the concentration peak would appear in the triangular vortex zone. (5) After ventilating for 750 s and 900 s, the peak value of the CO mass fraction was 157 mg/m^3^ and 51 mg/m^3^, which did not meet the limit concentration standard of 30 mg/m^3^. This might be caused by the combined effect of high altitude and the vortex zone. However, construction workers are permitted to inspect the working face for a limited period of time. The above conclusions are consistent with the analysis of the airflow field.

#### Analysis of case 2

Due to the actual schedule of tunnel construction, there is a situation that only one direction of the tunnel working face is excavated in a certain period of time. If the flow field remains unchanged, distinct initial distributions of CO will generate different variations in the concentration field.

**Figure 7:**
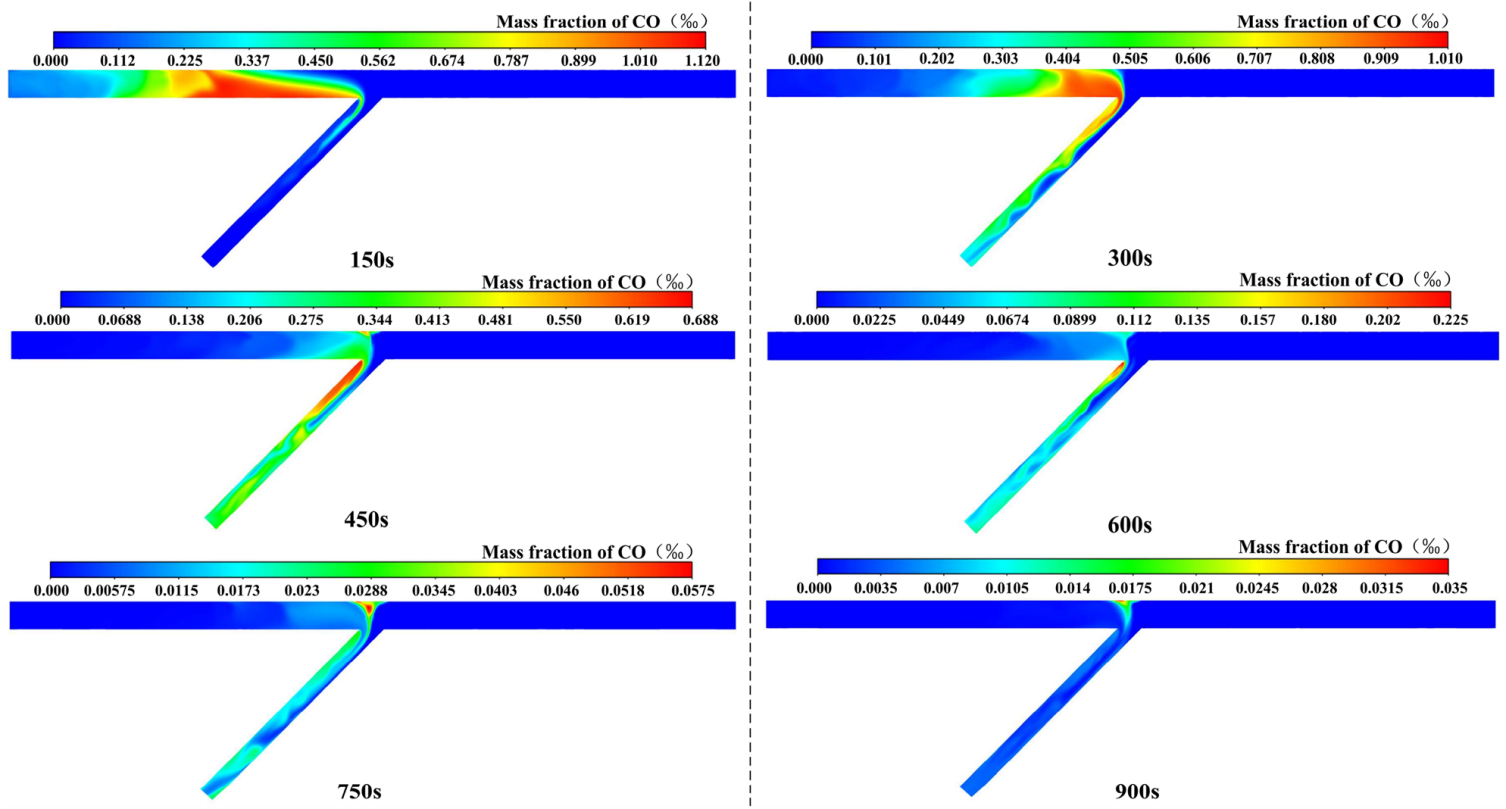
CO distribution of case 2.

The CO distribution over time of case 2 is shown in Fig. 7. The following conclusions can be drawn: (1) In the confluence, vortex, and multi-direction region of the inclined shaft, CO was distributed on the left side of the inclined shaft. This indicated that the airflow from the right tunnel was able to effectively prevent CO from diffusing to the right tunnel. (2) After ventilating for 450 s and 600 s, the peak concentration always appeared in the ventilation dead zone on the left side of the inclined shaft. CO would accumulate in this area for a long time. Because the high velocity air flow on the right side of the inclined shaft was not fully utilized, the ventilation efficiency of the whole area was reduced. (3) After ventilation for 750 s, the concentration peak appeared in the triangular vortex area. And after ventilation for 900 s, the peak concentration was 28 mg/m^3^, which reached the safe concentration standard of 30 mg/m^3^. (4) The value was not a simple multiple relationship when compared to the peak value of concentration after full mixing of airflow in both directions. This was mostly influenced by the vortex region and the velocity gradient distribution.

#### Analysis of case 3

**Figure 8:**
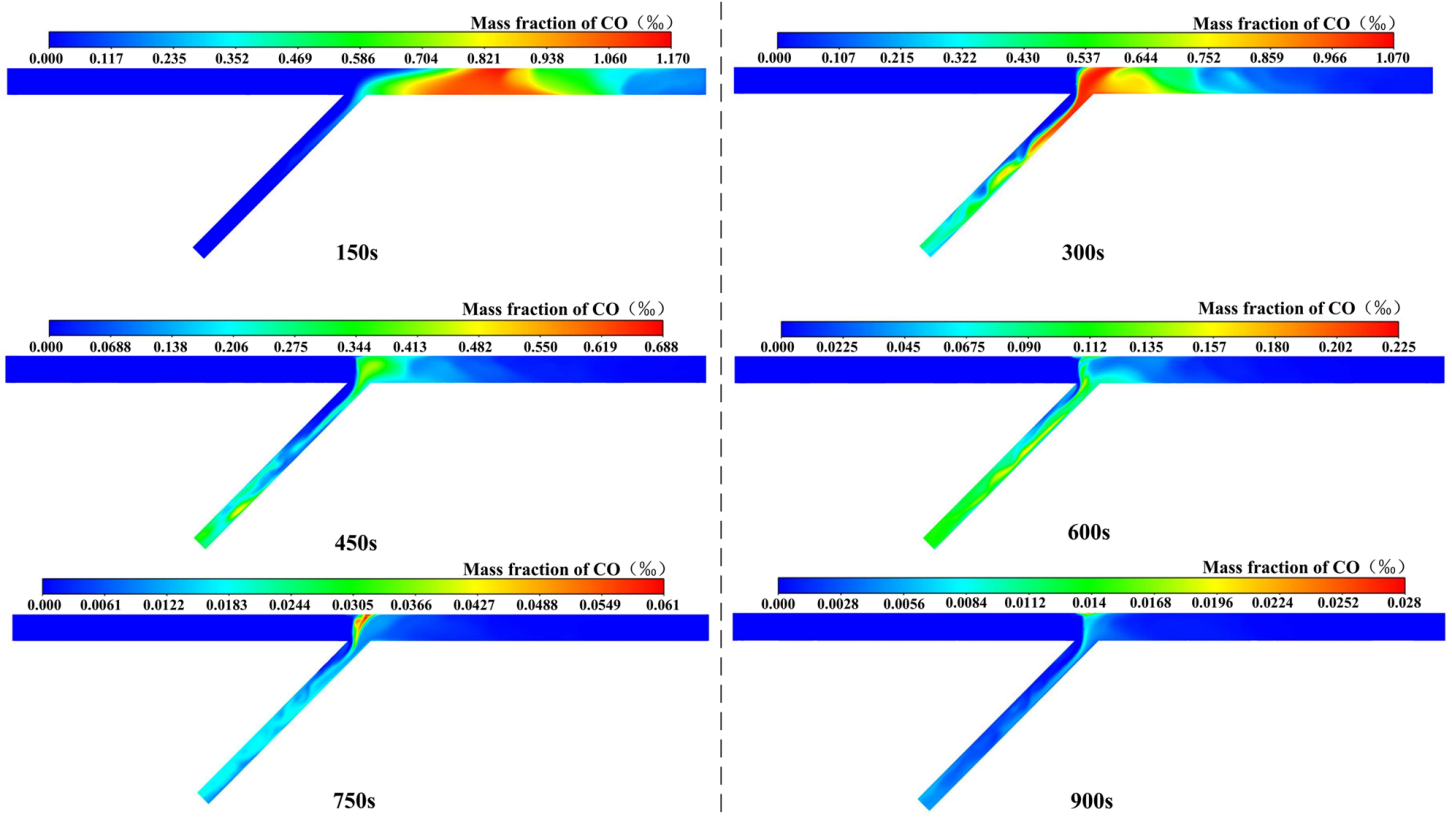
CO distribution of case 3.

The CO distribution over time of case 3 is shown in Fig. 8. The following conclusions can be drawn: (1) After ventilating for 150 s, the concentration peak of the right tunnel was greater than that of case 2. This was mostly due to the fact that the airflow from the right tunnel entered the inclined shaft across a large region, resulting in a slow flow rate, and the CO concentration was higher in the tunnel region. (2) Similar to case 2, the airflow from the left tunnel was able to effectively prevent CO from diffusing to the left tunnel and the ventilation dead zone on the left side of the inclined shaft. The difference was that in the multi-directional zone, CO was gradually evenly distributed in the inclined shaft. (3) After ventilating for 450 s and 600 s, the concentration peaks were smaller than those of case 2. This was due to the high airflow velocity on the right side of the inclined shaft. (4) Although the peak concentration appeared in the triangular vortex region after ventilating for 750 s and the value was slightly larger than in case 2, the peak concentration was 23 mg/m^3^ after ventilating for 900 s, which was significantly lower than that of case 2 and also reached the safety concentration standard of 30 mg/m^3^. The ratio of the peak concentration difference value to the larger concentration value was used to calculate the ventilation efficiency change. It showed that the overall ventilation efficiency was increased by 18% compared with case 2.

#### Analysis of case 4

**Figure 9:**
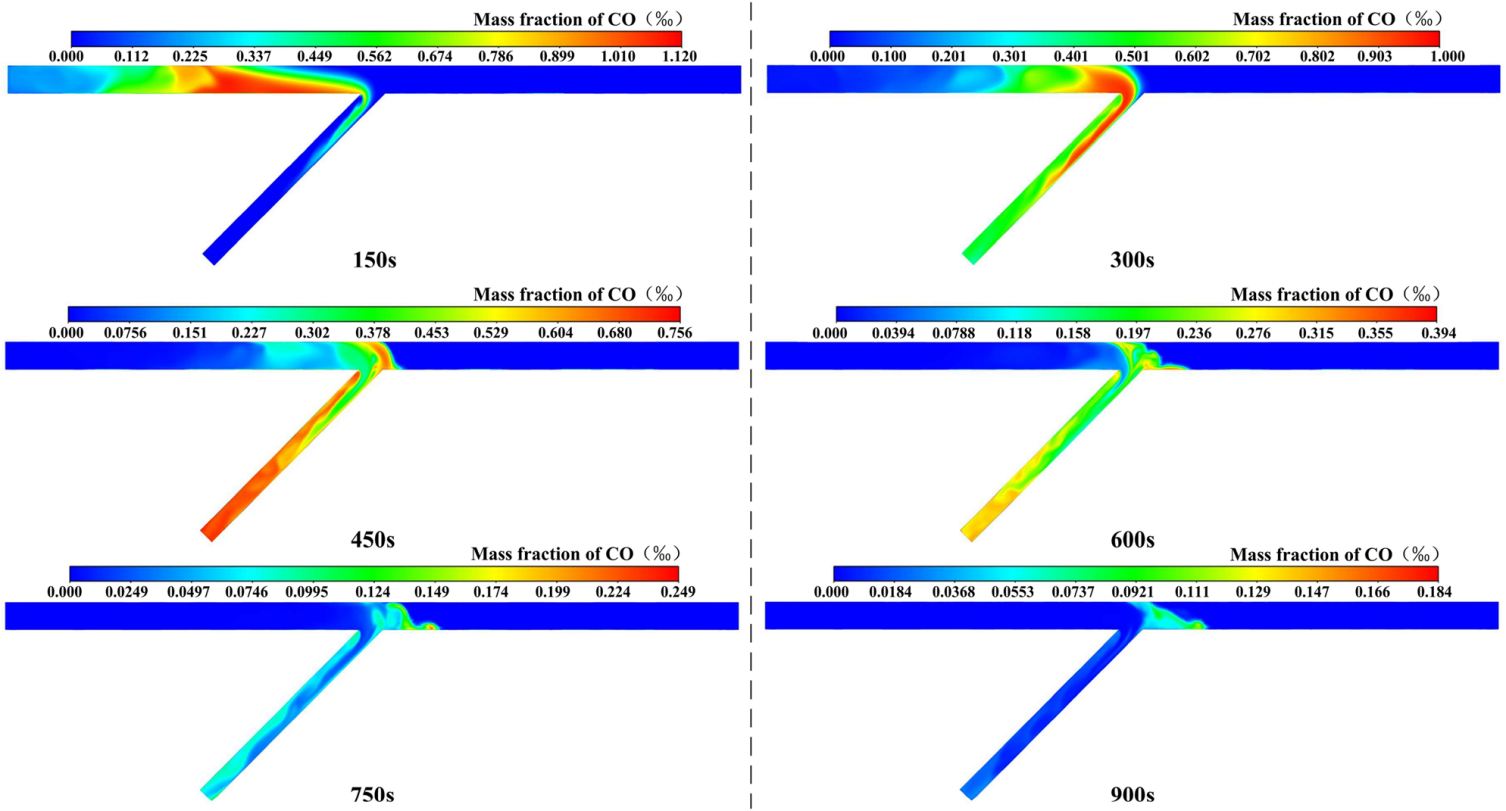
CO distribution of case 4.

If the excavation distance of the tunnel is less than 200 m, all construction personnel in the tunnel area should be evacuated. When there is only one working face for excavation, only the fan in this direction is turned on for energy savings, while the fans in the opposite direction are not. The CO distribution over time of case 4 is shown in Fig. 9. The following conclusions can be drawn: (1) After ventilating for 150 s and 300 s, the inclined shaft section was fully used for ventilation. Due to inertia, CO would first be close to the right wall of the inclined shaft, and then diffuse to the dead ventilation zone on the left side of the inclined shaft. (2) After ventilating for 450 s, a large area of high concentration would appear, and the peak concentration was greater than in the previous three cases, which is because the average wind speed of the inclined shaft section became smaller under the operation of a single fan. Meanwhile, CO would gradually begin to move to the right tunnel. (3) Due to the low wind speed, CO was distributed obliquely along the sidewall near the inclined shaft, and the diffusion range in the right tunnel did not change much.

#### Analysis of case 5

**Figure 10:**
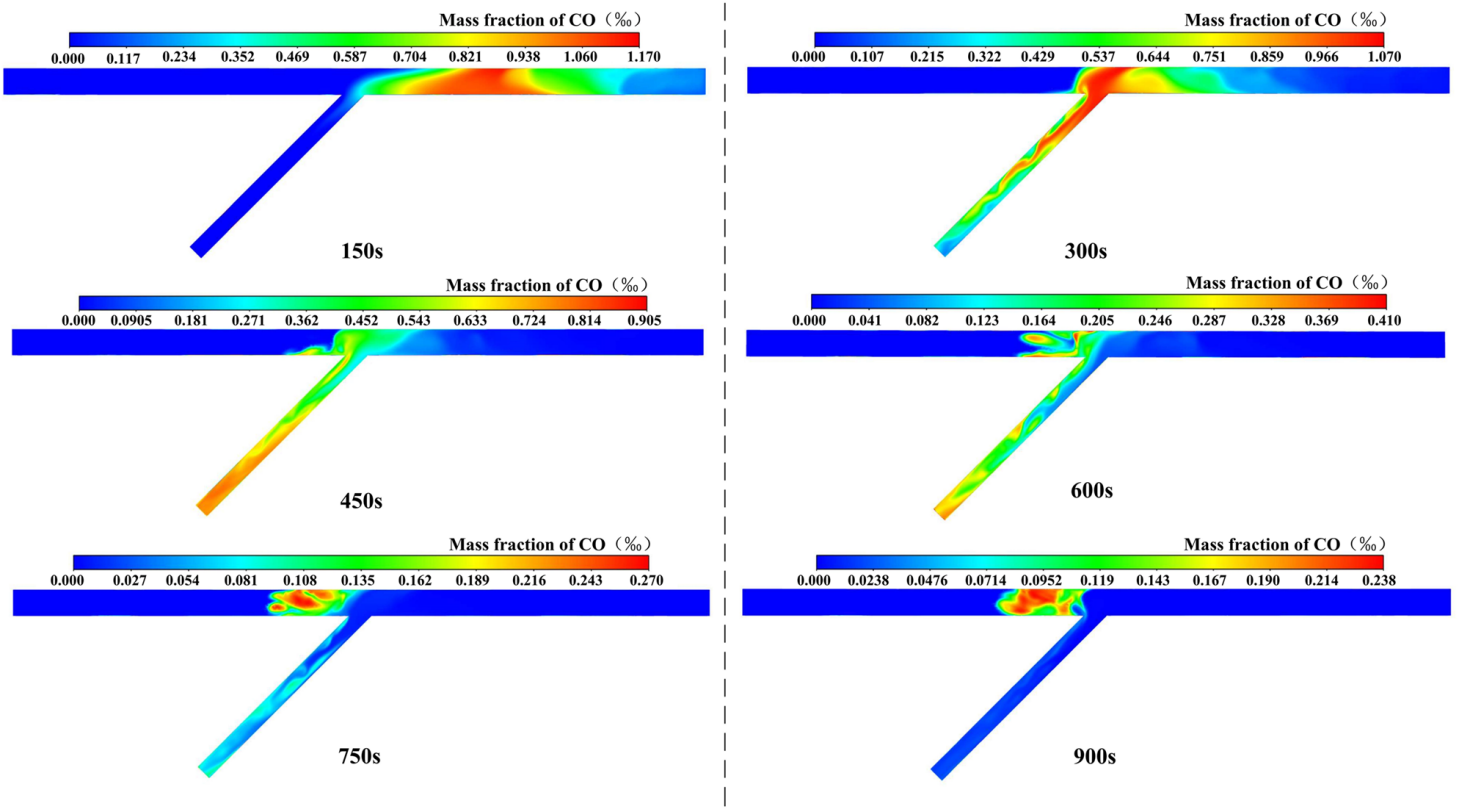
CO distribution of case 5.

The CO distribution over time of case 5 is shown in Fig. 10. The following conclusions can be drawn: (1) After ventilating for 300 s, CO had diffused to the left tunnel. It was considered that when the wind came only from the right tunnel, the velocity distribution was relatively average. CO would not be distributed in a slim strip during migration to the inclined shaft, causing more CO to diffuse into the left tunnel. (2) Due to the formation of a vortex region in the left tunnel, an obvious concentration peak region would be formed in the left tunnel. (3) After ventilating for 750 s and 900 s, the peak value of the CO mass fraction was 219 mg/m^3^ and 193 mg/m^3^, which did not meet the limit concentration standard of 30 mg/m^3^. (4) In comparison to case 4, the peak CO concentration was higher, the distribution range was wider, and the ventilation time was longer. To ameliorate this condition and increase ventilation efficiency, consider installing a jet fan or an air curtain.

### A two-stage ventilation scheme

Before re-entering the opposite direction for construction, the fan should be turned on for ventilation first. Taking case 4 as an example, CO just moved from the left tunnel to the right tunnel after ventilating for 600 s, as shown in Fig. 9. At this moment, the fans in both directions were activated simultaneously for ventilation to stop CO from diffusing in the other direction. In the next 300 s, the ventilation effect was simulated and compared with case 2.

**Figure 11:**
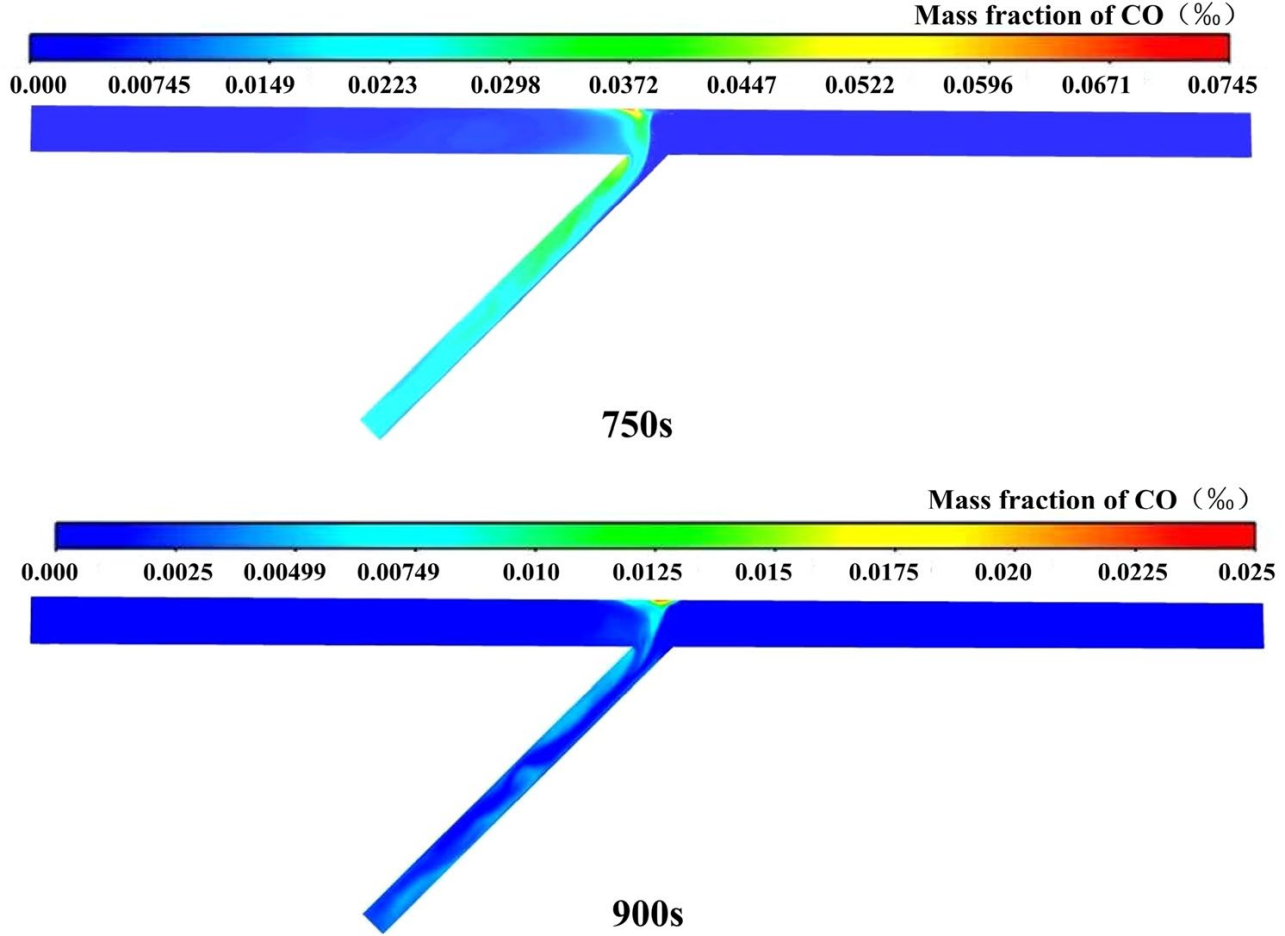
CO distribution of two-stage ventilation scheme.

The CO distribution over time of two-stage ventilation scheme is shown in Fig. 11. After ventilating for 750 s and 900 s, the peak value of the CO mass fraction was 59.6 mg/m^3^ and 20 mg/m^3^, which was lower than that of case 2 and also reached the safety concentration standard of 30 mg/m^3^. Assuming that the power of all fans is the same, the product of power and ventilation time is used to represent the energy consumption. And the ratio of the energy consumption difference value to the larger energy consumption value is used to calculate the energy consumption change. The ventilation efficiency was increased by 28.5% and the energy consumption was reduced by at least 33% compared with case 2.

Therefore, a two-stage ventilation scheme was proposed when constructing a single working face. Firstly, turn on the fan in the direction of the excavation working face. And then, turn on the fans in both directions at the same time. This ventilation scheme has the potential to reduce energy consumption.

## Conclusions

The airflow field and CO concentration field characteristics in high-altitude tunnel by inclined shaft were analyzed mainly through a three-dimensional numerical model. The effects of different fan opening modes and different initial CO concentration distributions on the ventilation were discussed. And a new optimized ventilation scheme was proposed to improve the ventilation efficiency and reduce energy consumption. The following are the main conclusions.

1. The main difference in the ventilation wind field was reflected in the position of the vortex region, which was directly related to ventilation efficiency. The vortex region caused CO to remain for an extended period of time and increased energy consumption.
2. Various initial CO concentration distributions showed different migration when two working faces were under construction at the same time. The CO concentration could achieve the safe level after ventilating for 15 min when a single working face was constructed. Eliminating vortex zones and fully using high velocity airflow of inclined shaft could improve relative ventilation efficiency by at least 18%.
3. The distribution of CO concentration would alter as a result of different fan opening modes. Only one of the working faces was under construction and the fan in this direction was turned on, the CO would diffuse to the tunnel in the other direction, forming a high concentration peak area.
4. The two-stage ventilation scheme should be adopted when only one of the working faces was under construction. The ventilation efficiency was increased by 28.5% and the energy consumption was reduced by at least 33%.

The impacts of altitude, ventilation technique, building method, and air duct layout on ventilation should be completely studied for the future study. At the same time, this work primarily used numerical simulation as a research method. The reasonableness of numerical simulation should be proved more precisely in the next steps, such as on-site monitoring, model testing, and so on.

## Data Availability

The datasets generated during and/or analyzed during the current study are not publicly available but are available from the corresponding author on reasonable request.
